# *Amaranthus* grain as a new ingredient in diets for dairy cows: productive, qualitative, and *in vitro* fermentation traits

**DOI:** 10.1002/jsfa.11761

**Published:** 2022-01-22

**Authors:** Serena Calabrò, Marianna Oteri, Alessandro Vastolo, Monica Isabella Cutrignelli, Massimo Todaro, Biagina Chiofalo, Fabio Gresta

**Affiliations:** ^1^ Department of Veterinary Medicine and Animal Production (DMVPA) University of Napoli Federico II Naples Italy; ^2^ Department of Veterinary Sciences University of Messina, Polo Universitario Annunziata Messina Italy; ^3^ Department of Agricultural, Food and Forest Science (SAAF) University of Palermo Palermo Italy

**Keywords:** *Amaranthus cruentus*, *Amaranthus hypochondriacus*, seed yield, quality traits, rumen fermentation, volatile fatty acids

## Abstract

**BACKGROUND:**

In recent decades, grain amaranths have attracted attention due to their valuable combination of nutritional traits, with higher protein and oil content than conventional cereals. Before they can be proposed as an unconventional ingredient in animal feed, many aspects still need to be investigated from field production to nutritive value. The present research aimed to study the agronomic traits, proximate composition, and digestibility/degradability, fatty acid profile, antioxidant activity, and total phenolic content of two grain amaranth species, *Amaranthus cruentus* and *Amaranthus hypochondriacus* (for a total of six accessions), grown in a Mediterranean environment.

**RESULTS:**

Both species showed seed yields comparable to or higher than the traditional cereal crops in the same environment. On the whole, *A. cruentus* resulted in a higher seed production than *A. hypochondriacus*. Mexico and Montana accessions, both belonging to *A. cruentus*, showed the highest yield (3.73 t ha^−1^, on average). Few differences emerged in nutritive value between species and accessions: the Illinois accession of *A. cruentus* showed the best performance in terms of *in vitro* degradability and gas production, but not for volatile fatty acid production; the fermentation kinetics was slowest in the Illinois accession and fastest in the Montana accession of *A. cruentus* and the India accession of *A. hypochondriacus*.

**CONCLUSION:**

From a health perspective, the Nebraska accession of *A. hypochondriacus* represents the best accession, with the lowest saturated fatty acid content and the highest polyunsaturated fatty acid content. © 2022 The Authors. *Journal of The Science of Food and Agriculture* published by John Wiley & Sons Ltd on behalf of Society of Chemical Industry.

## INTRODUCTION

In recent decades, a reduction in biodiversity and in the number of crops for food and feed use has stimulated researchers to study alternative underexploited crops with nutritional and nutraceutical potential. Among these, grain amaranths (*Amaranthus* spp.) can be considered an interesting unconventional group of crops due to the high nutritional and nutraceutical value of their seeds, and to their ability to germinate and grow in unfavorable environmental conditions.^1^, ^2^, ^3^, ^4^ Until a few decades ago, amaranth species were considered invasive weeds, but in recent years they have gained importance and now they are considered pseudo‐cereals. The grains exhibit a valuable combination of nutritional traits, with a higher crude protein (CP: 130–180 g kg^−1^) and ether extract (EE: 63–81 g kg^−1^) content^5^, ^6^ than conventional cereals such maize (CP: 100 g kg^−1^ and EE: 45 g kg^−1^), wheat (CP: 140 g kg^−1^ and EE: 26 g kg^−1^), rice (CP: 85 g kg^−1^ and EE: 42 g kg^−1^),^7^, ^8^ and sorghum (CP: 120 g kg^−1^ and EE: 40 g kg^−1^)^9^ along with a qualified fatty acid profile comprising about 360 g kg^−1^ of saturated fatty acids (SFA) and 640 g kg^−1^ of unsaturated fatty acids (UFA), the latter mainly represented by oleic (320–330 g kg^−1^) and linoleic (270–280 g kg^−1^) acids.^5^, ^6^ Moreover, amaranth contains interesting secondary metabolites, which contribute to its potential health benefits,^10^ such as phenolic compounds, which are considered to be cholesterol‐lowering, anti‐thrombosis, anti‐inflammatory, anti‐cancer factors,^10^ and a potential source of dietary antioxidants.^5^, ^6^ Nowadays, grain amaranth includes a large group of species; among these, *Amaranthus hypochondriacus* and *Amaranthus cruentus*, both natives of Central America, show promising results as feedstuff for ruminants, although many aspects still need to be investigated. In fact, the research on the characterization of productive and qualitative traits of *A. hypochondriacus* and *A. cruentus* to be used in animal diet is scarce and fragmentary. A few authors^11^, ^12^ have studied the effect of *A. hypochondriacus* grain as a partial substitute for barley in ruminant diets, obtaining valuable results. *Amaranthus cruentus* is also considered an interesting crop from a nutritional and nutraceutical point of view for animal feeding.^13^ However, as far as we are aware, no information has been reported in the literature about the *in vitro* rumen fermentation parameters.

As Mediterranean cropping systems suffer from a chronic shortage of quality products for animal nutrition,^14^ the identification of a valuable grain crop with chemical and fermentation characteristics that meet the nutritional requirements of ruminants is of considerable interest. This suggests that a holistic approach including both field cultivation and laboratory assays should be promoted.

The present research aimed to study the agronomic traits, proximate composition, *in vitro* digestibility/degradability, fatty acid profile, antioxidant activity, and total phenolic content of two amaranth species, *A. cruentus* and *A. hypochondriacus*, grown in a Mediterranean area. The hypothesis is that grain amaranth could represent a possible source of nutrients and bioactive compounds in ruminant diets.

## MATERIALS AND METHODS

### Plant material

Three accessions of two amaranth species, *A. cruentus* (origin: Mexico, Montana, Illinois; plant inventory: 477913, 538255, 606797, respectively) and *A. hypochondriacus* (origin: India, Nebraska, Pennsylvania; plant inventory: 477915, 558499, 572256, respectively) were compared in this trial. The experimental field trial was carried out in two phases: in the first phase, amaranth seeds were grown in a nursery, while in the second phase plants were transplanted in the field. The seeds of the two species, obtained from the United States Department of Agriculture (USDA) seed bank (Washington, DC, USA), on March 21, 2014, were manually sowed inside a nursery (kept at 26 °C and 85 ± 5% RH) in expanded polystyrene trays. When the fourth true leaf appeared, plants were transplanted in a field with a density of 10 plants per m^2^ (1.0 × 0.10 m) in Bovalino (RC) (20 m a.s.l. 38° 08′ N, 16° 10′ E, Calabria, Italy) in a soil with a sandy‐loam texture. A randomized block design with plots of 9 m^2^ (3 × 3 m) three‐time replicated was adopted.

Before the transplant, the soil was shallow plowed and fertilized with 40 kg ha^−1^ of N, 80 kg ha^−1^ of P_2_O_5_, and 60 kg ha^−1^ of K_2_O. A further supply of 80 kg ha^−1^ of N, as ammonium nitrate, was broadcasted before anthesis. During the trial, weeds were hand weeded, and a total volume of 3200 m^3^ ha^−1^ was supplied with a drip system during the crop cycle. Seed harvest was staggered from 29 June to 11 July in relation to the degree of maturation of the different accessions. Then seeds were separated with a laboratory thresher.

The average temperature during the trial ranged from 17.7 °C, recorded at the end of April, to 25.2 °C, recorded at the end of June (lowest value: 14.6 °C; highest value 30.1 °C). As usual, in the summer Mediterranean climate, rainfalls during the trial were inconsistent (26.4 mm).

Before all analyses, the seeds were ground to pass a 1.1 mm screen (SM 100, Retsch, Haan, Germany).

### Chemical analysis

Moisture and ash content measurements in amaranth ground seeds (2 g) were performed using a TGA‐701 thermogravimetric analyzer purchased from LECO Corporation (Milano, Italy). The automated instrument measures weight loss as a function of temperature in a controlled environment for the determination of total moisture and ash, until a constant weight is reached. For the moisture measurement, the sample was dried to constant weight at 103 °C.^15^ For the ash content it was incinerated to constant weight at 600 °C.^16^

Nitrogen content was determined using the Kjeldahl procedure^16^ by using a Kjeltec system, provided by FOSS (Padua, Italy). Crude protein (CP) content was determined using the Kjeldahl procedure16 by using a Kjeltec system, provided by FOSS (Padua, Italy). The lipid content (EE) of the six accessions of *Amaranthus* seeds was determined according to the official method^17^ using the Soxtec™ 8000 Extraction and Hydrotec™ 8000 Hydrolysis Systems supplied from FOSS (Padua, Italy). Before the lipid extraction, each sample was hydrolyzed with HCl (3 N) in the Hydrotec system.

The fibrous fractions, such as neutral detergent fiber using heat‐stable amylase and exclusive of residual ash (aNDFom), ash‐free acid detergent fiber (ADFom), and acid detergent lignin (ADL), were determined following the AOAC^18^ and Van Soest *et al*.^19^ methods. The determination of fibrous fractions was carried out gravimetrically.

Total starch was determined using a Megazyme Total Starch Assay Kit (Megazyme©, NEOGEN, Lansing, Michigan, USA) according to AOAC method 996.11.^18^ The analysis was performed using the amyloglucosidase/*α*‐amylase method and spectrophotometric quantification was carried out using a UV‐2600 (UV‐visible spectrophotometer) from Shimadzu (Milan, Italy). The absorbance was measured at a wavelength of 510 nm.

Each chemical analysis was performed in duplicate.

### Analysis of fatty acids and quality index calculation

Direct transesterification of lipids was carried out for fatty acid (FA) analysis via acid methylation of the extracts with 2 mL of a methanol:sulphuric acid (9:1, v/v) mixture for 1 h at 100 °C.^20^ Fatty acid methyl esters (FAMEs) were dissolved with 1 mL of *n*‐hexane, filtered using nylon filters 0.45 μm and injected into a gas chromatography (GC) system with a flame ionization detector (FID) for FAME analysis, as reported in previous papers.^5^, ^6^

A quali‐quantitative analysis of FAMEs was performed in duplicate by using a GC‐FID (TRACE 1310) system provided with an AI 1310 Autoinjector/AS 1310 Autosampler from Thermo Fisher Scientific (Milan, Italy). Chromatographic separation of FAMEs was performed on an Omegawax 250 (Supelco, Bellefonte, PA, USA), 30 m × 0.25 mm, 0.25 μm film thickness (*d*_*f*_) capillary column using the following GC conditions: oven temperature program: 0–5 min, 100 °C; 5–40 min, 100–240 °C (4 °C min^−1^); 40–60 min, 240 °C; injection volume, 0.5 μL; split ratio, 1:50; injector and detector temperature, 250 °C; carrier gas (He) at a flow rate of mL min^−1^; make‐up gas (N_2_) flow, 40 mL min^−1^; H_2_ flow, 35 mL min^−1^; airflow, 350 mL min^−1^. Data acquisition was processed using the Chromeleon™ Data System Software (Version 7.2.9) from Thermo Fisher Scientific (Milan, Italy). The FAs of the amaranth samples were identified, comparing the relative retention times of FAMEs with those of a standard mix solution (mix 37 FAMEs, Supelco, Inc., Bellefonte, PA, USA) under the same analytical conditions. The FA concentrations were expressed as g kg^−1^, where 1 kg was the total of all areas of the identified FAMEs.

The peroxidation index (PI) was calculated using Luciano *et al*.’s^21^ equation, Eqn (1):

(1) $$\mathrm{PI}=\left(\text{dienoic}\times 1\right)+\left(\text{trienoic}\times 2\right)+\left(\text{tetraenoic}\times 3\right)+\left(\text{pentaenoic}\times 4\right)+\left(\text{hexaenoic}\times 5\right)$$

### Total phenolic analysis

The total phenolic content (TPC) of milled amaranth seeds was determined using the method described by López‐Mejía *et al*.^22^ This consisted of methanol treatment of about 10 g of the sample and gravimetric determination after evaporating the solvent. The TPC was determined spectrophotometrically after the reaction with Folin–Ciocalteu reagent (FCR) and incubation in a dark environment at room temperature following the Karamać *et al*. method.^23^ The absorbance was measured at a wavelength of 725 nm and the results were expressed as mg of gallic acid equivalents per gram (GAE) (mg g^−1^) of sample.^5^ Each determination was performed in duplicate.

### Antioxidant activity analysis

Methanol extracts of polyphenols were analyzed for their antioxidant capacity using a 2,2‐diphenyl‐1‐picrylhydrazyl (DPPH^•^) assay. The DPPH^•^ scavenging activity was evaluated in duplicate on milled amaranth seeds following the method reported by Brand‐Williams *et al*.^24^ An aliquot of methanol solution (0.1 mL) containing different concentrations (from 2 to 10 mg mL^−1^ of initial sample) was added to 0.25 mL of 1 mM DPPH^•^ and 2 mL of methanol, mixed, and the absorbance was measured after 20 min (λ = 517 nm). Data were used to determine the quantity (μg) of polyphenols needed to scavenge 50% of the DPPH^•^ (EC_50_). Then, EC_50_ values, considered as the dried extract concentration (mg mL^−1^ solution) needed to scavenge the 50% of initial DPPH^•^ were evaluated.

### *In vitro* gas production

All samples (three accessions for the two varieties) were evaluated by an *in vitro* gas production technique^25^ at the feed evaluation laboratory of the Department of Veterinary Medicine and Animal Production, University of Napoli Federico II, Italy. For each accession, three serum flasks (120 mL) were incubated with the samples (1 ± 0.0029 g) with the anaerobic medium (74 mL) and ruminal *inoculum* (10 mL) at 39 °C, under anaerobic conditions. For the *inoculum* preparation, the ruminal fluid was collected at an authorized slaughterhouse^26^ from six fasting young bulls (*Bos taurus*) fed a standard diet (DM) (g kg^−1^: NDF 461 and CP 150). The collected rumen content was transported rapidly to the laboratory in a pre‐warmed thermos, where it was pooled, filtered through a cheesecloth, and added to each bottle. During the period of incubation (48 h) the gas production of the fermenting cultures was recorded (from 2 to 24 h intervals) using a manual pressure transducer (Cole and Palmer Instrument Co., Vernon Hills, IL, USA). The fermentation was stopped by cooling the bottles at 4 °C and the fermentation liquor was analyzed for pH using a pH meter (ThermoOrion 720 A+, Fort Collins, CO, USA) and sampled for the end‐product analysis. The extent of sample disappearance, expressed as organic matter degradability (OMD, %), was determined by the difference between the incubated OM and the residual matter after filtration through sintered glass crucibles (Schott Duran, Mainz, Germany, porosity # 2) and burning at 550 °C for 3 h. The cumulative gas volume (OMCV, mL g^−1^) was related to the incubated organic matter, and the gas profiles were fitted to the exponential model described by Groot *et al*.^27^ as shown in Eqn (2):

(2) $$G=\frac{A}{1+{\left(\frac{B}{t}\right)}^{C}}$$

where *G* is the total gas produced (mL g^−1^ of incubated OM) at *t* (h) time, *A* is the asymptotic gas production (mL g^−1^ of incubated OM), *B* is the time at which half of the asymptote is reached (h), and *C* is the switching characteristic of the curve. The time of maximum fermentation rate (*T*_max_, h) and maximum fermentation rate (*R*_max_, mL h^−1^) were also calculated^28^ as follows:

(3) $$T_{\max}=C*{\left(\frac{B-1}{B+1}\right)}^{1/B}$$

(4) $$R_{\max=}\frac{A*B^{C}*B*{T_{\max}}^{\left(B-1\right)}}{1+{\left(C^{B}*{T_{\max}}^{-B}\right)}^{2}}$$

For the volatile fatty acid (VFA) (mmoL g^−1^) analyses, samples of fermenting liquor from each bottle were centrifuged twice at 12 000×*g* for 10 min at 4 °C (Universal 32R centrifuge, Hettich FurnTech Division DIY, Melle‐Neuenkirchen, Germany); then 1 mL of supernatant was diluted in 1 mL of oxalic acid (0.06 mol L^−1^) and injected into a gas chromatograph (ThermoQuest 8000top Italia SpA, Rodano, Milan, Italy) equipped with a fused silica capillary column (30 x 0.25 mm, 0.25 μm film thickness, Supelco, Inc., Bellefonte, PA, USA). Each fatty acid (acetate, propionate, iso‐butyrate, butyrate, valerate, and iso‐valerate) concentration was measured comparing the sample peak areas of each VFA with that the external standard.^29^ Branched‐chain fatty acids proportions (BCFAs) were also calculated as in Eqn (5):

(5) $$\left[\left(\mathrm{iso}-\text{valerate}+\mathrm{iso}-\text{butyrate}\right)/\mathrm{VFA}\right]\times 100$$

### Statistical analysis

All parameters obtained were statistically analyzed to detect the differences between accessions by a one‐way ANOVA according to the model in Eqn (6):

(6) $$y_{i}=\mu +{\mathrm{acc}}_{i}+\varepsilon_{i}$$

where *y* is the experimental data, *μ* represents the general mean, acc is the effect of accession (*i* = 1, 2, …, 6), and *ε* is the error term. The significance level was verified using the HSD Tukey test.

The correlations between chemical composition values and fermentation parameters and between *in vitro* fermentation and fatty acid profile, peroxidation index, total phenolic compounds and antioxidant activity, were calculated. All the statistical analysis were performed using the JMP® Version 14 SW software (SAS Institute Inc., Cary, NC, USA, 1989–2019).

## RESULTS

### Agronomic data

**Figure 1:**
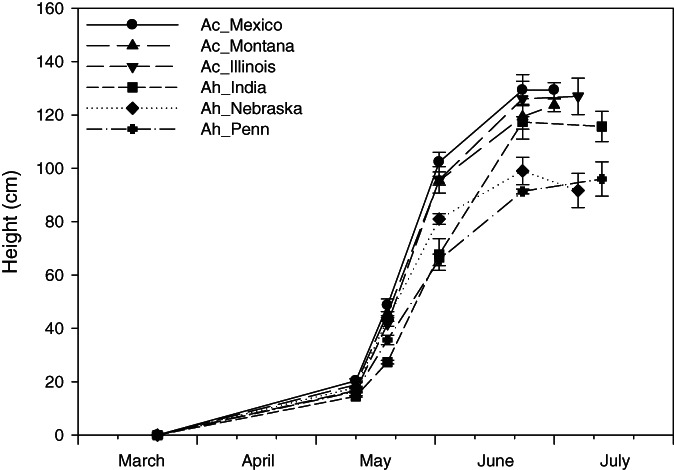
Height of the accessions of the two amaranth species during the trial. Ac: *A. cruentus*, Ah: *A. hypochondriacus*.

All amaranth plants reached the greatest height at harvest in July, showing the most rapid growth from the end of May to the middle of June (Fig. 1). At harvest, the three accessions of *A. cruentus* showed a plant height ranging from 117 to 140 cm with an average of 127 cm, while *A. hypochondriacus* reached a shorter height ranging from 86 to 127 cm, with an average of 101 cm.

**Figure 2:**
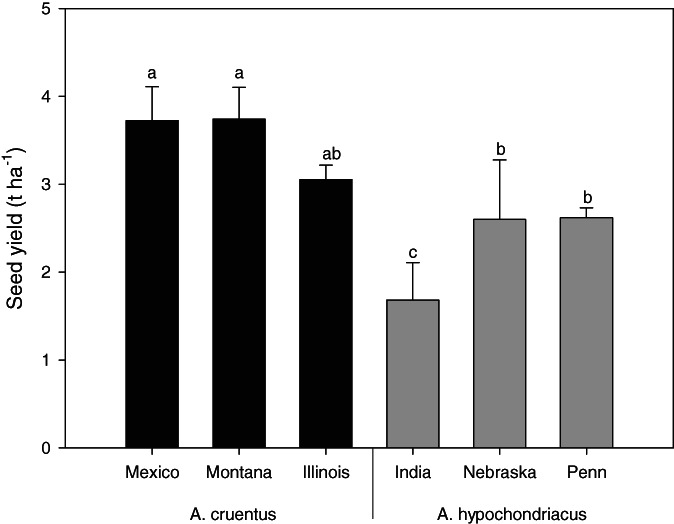
Seed yield of the accessions of the two amaranth species. Different letters indicate significant differences (*P* < 0.05).

The three accessions of *A. cruentus*, Mexico, Montana, and Illinois, proved to be the most productive accessions with an average seed yield of 3.48 t ha^−1^ (*P* < 0.05) (Fig. 2). On the other hand, the India accession of *A. hypochondriacus* showed the lowest yield value (1.68 t ha^−1^).

**Figure 3:**
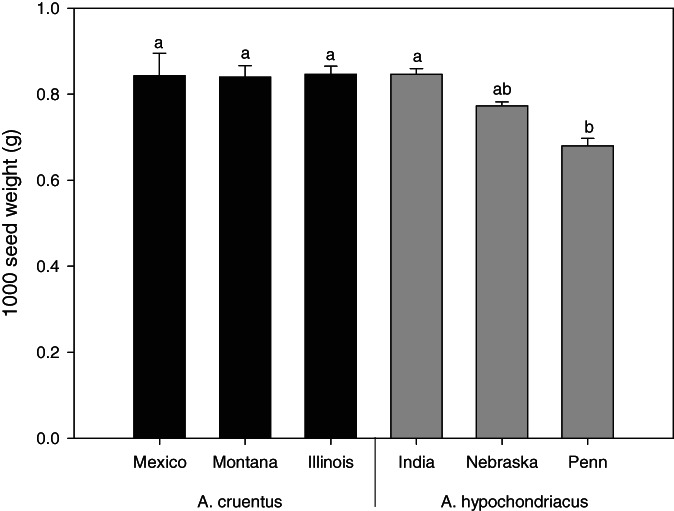
One thousand seed weight of the accessions of the two amaranth species. Different letters indicate significant differences (*P* < 0.05).

The thousand seed weight showed that three accessions of *A. cruentus* plus India of *A. hypochondriacus* (0.84 g, on average) were significantly higher in comparison with the Pennsylvania accession of *A. hypochondriacus* (0.68 g) (*P* < 0.05) (Fig. 3).

### Chemical composition

Table 1 shows the results of chemical composition for the six accessions of amaranth seed. Except for aNDF and ADL, significant differences emerged among accessions, even if they did not show a clear trend. In particular, the Mexico accession showed the highest (*P* < 0.01) values for EE. Montana and Illinois presented the lowest values of crude protein (*P* < 0.01). The Illinois accession also showed the lowest (*P* < 0.01) amount of ash, and the highest aNDFom content, even though the difference was not statistically significant. The Montana accession showed the lowest ADFom content. In the Montana and Illinois accessions, starch values were the highest, whereas in Nebraska the lowest (*P* < 0.01). Nebraska and India showed the highest value of ash (*P* < 0.01).

**Table 1:** Chemical composition (g kg^−1^, a.f.) of the six accessions of amaranth seeds (*n* = 12)

Species	Accession	DM	Ash	EE	CP	aNDFom	ADFom	ADL	Starch
*Amaranthus cruentus*	Mexico	898^A^	27^DE^	77^A^	165^A^	92	55^ABC^	22	529^B^
	Montana	897^A^	28^CD^	70^B^	148^B^	84	49^C^	21	583^A^
	Illinois	891^B^	25^E^	70^B^	146^B^	100	54^BC^	21	536^A^
*Amaranthus hypochondriacus*	India	897^A^	30^AB^	49^D^	164^A^	89	55^ABC^	20	512^C^
	Nebraska	898^A^	32^A^	64^C^	158^A^	97	60^A^	22	504^D^
	Pennsylvania	899^A^	29^BC^	62^C^	160^A^	98	58^A^	21	515^C^
RMSE		0.05	0.02	0.11	0.33	2.88	0.25	0.09	7.08

DM: dry matter; CP: crude protein; EE: ether extract; aNDFom: neutral detergent fiber; ADFom: acid detergent fiber; ADL: acid detergent lignin. RMSE: root mean square error. Down the column upper case and lower case letters indicate *P* < 0.01 and *P* < 0.05, respectively.

### Fatty acids, total phenolic contents, and antioxidant activity

As shown in Table 2, significant differences emerged among accessions with regard to the fatty acid profile. Among the fatty acids of nutritional interest, the Mexico accession showed the lowest (*P* < 0.01) level of myristic acid (C14:0), stearic acid (C18:0) and, together with the Nebraska accession, of palmitic acid (C16:0). The Montana accession showed the lowest (*P* < 0.01) level of *α*‐linolenic acid (C18:3n3) and, together with the Illinois accession, the highest (*P* < 0.01) amount of oleic acid (C18:1n9). The Illinois accession showed the highest (*P* < 0.01) level of myristic acid and stearic acid and the lowest (*P* < 0.01) amount of linoleic acid (C18:2n6). The India accession showed the highest (*P* < 0.01) level of *α*‐linolenic acid and, together with the Nebraska accession, of linoleic acid whereas, together with the Pennsylvania accession, it displayed the lowest (*P* < 0.01) level of oleic acid. Finally, the Pennsylvania accession showed the highest (*P* < 0.01) level of palmitic acid.

**Table 2:** Fatty acid profile (g kg1^−1^) in the six accessions of amaranth seeds (*n* = 12)

Species	Accession	C14:0	C16:0	C16:1	C17:0	C18:0	C18:1n9	C18:1n7	C18:2n6	C18:3n3	C20:0	C22:0	Others
*Amaranthus cruentus*	Mexico	4.2^E^	258^C^	2.4^b^	4.9^B^	44^F^	324^B^	14^B^	308^B^	4.7^BC^	7.4^CD^	3.8^CD^	25^A^
	Montana	7.0^D^	275^B^	2.6^b^	4.7^AB^	52^D^	344^A^	15^B^	266^C^	3.7^E^	8.4^BC^	4.0^BC^	18^C^
	Illinois	9.2^A^	268^B^	7.1^a^	3.6^D^	80^A^	360^A^	18^A^	228^D^	3.9^DE^	6.4^D^	3.2^E^	13^D^
*Amaranthus hypochondriacus*	India	7.5^C^	270^B^	2.4^b^	4.2^C^	59^C^	265^D^	14^B^	340^A^	6.3^A^	8.5^B^	4.2^B^	20^C^
	Nebraska	6.7^D^	253^C^	2.5^b^	4.1^C^	50^E^	292^C^	14^B^	343^A^	5.0^B^	7.5^BC^	3.6^D^	18^C^
	Pennsylvania	8.3^B^	290^A^	4.5^ab^	5.2^A^	64^B^	266^D^	14^B^	307^B^	4.4^CD^	10^A^	4.9^A^	22^B^
RMSE		0.001	0.64	0.11	0.001	0.01	1.76	0.01	0.69	0.001	0.007	0.0006	0.03

The concentration of fatty acids was expressed as g kg^−1^, considering 1 kg the sum of the areas of all FAME identified. C14:0 = myristic acid; C16:0 = palmitic acid; C16:1 = palmitoleic acid; C17:0 = heptadecanoic acid; C18:0 = stearic acid; C18:1n9 = oleic acid; C18:1n7 = cis‐vaccenic acid; C18:2n6 = linoleic acid; C18:3n3 = *α*‐linolenic acid; C20:0 = arachidic acid; C22:0 = behenic acid. RMSE: root mean square error. Down the column upper case and lower case letters indicate *P* < 0.01 and *P* < 0.05, respectively.

Table 3 shows the fatty acid classes and the quality index for the six accessions of amaranth seeds. Considering all the parameters studied, the trend as a whole is quite variable. Regarding the amount of SFA, the Pennsylvania accession showed the highest (*P* < 0.01) level whereas the Mexico and Nebraska accessions displayed the lowest (*P* < 0.01) level. Regarding MUFA, the Illinois accession showed the highest (*P* < 0.01) level while India and Pennsylvania had the lowest (*P* < 0.01). The level of PUFA was significantly higher in the India and Nebraska accessions (*P* < 0.01) and lower in the Illinois accession (*P* < 0.01). The India accession showed the highest (*P* < 0.01) amount of n3‐PUFA, and Montana had the lowest (*P* < 0.01) level. The level of n6‐PUFA was the highest (*P* < 0.01) in the India accession and Nebraska and the lowest (*P* < 0.01) in Illinois. Consequently, the SFA/PUFA ratio showed the highest (*P* < 0.01) value in Pennsylvania and the lowest (*P* < 0.01) level in Mexico and Nebraska. Concerning antioxidant activity, the peroxidation index showed the highest (*P* < 0.01) values in the India and Nebraska accessions and the lowest (*P* < 0.01) values in the Montana and Illinois accessions. Nebraska showed the highest (*P* < 0.01) TPC content whereas Montana showed the (*P* < 0.01) lowest value. As regards the antioxidant power, the DPPH^•^ values showed the lowest (*P* < 0.01) level in Pennsylvania in comparison with all the other accessions of both *A. cruentus* and *A. hypochondriacus*. The Illinois accession maintains a low DPPH^•^ value (high antioxidant activity) due to the high content of SFAs (Table 3). Indeed, as well known, the saturated fatty acids are more stable than the polyunsaturated ones.

**Table 3:** Fatty acid classes (g kg^−1^), ratios, total phenolic content (mg g^−1^) and antioxidant properties (mg mL^−1^) in the six accessions of amaranth seeds (*n* = 12)

Species	Accession	SFA	MUFA	PUFA	SFA/UFA	n3‐PUFA	n6‐PUFA	PI	TPC	DPPH^•^
*Amaranthus cruentus*	Mexico	322^D^	340^C^	313^B^	0.50^D^	4.7^BC^	308^B^	31.8^B^	0.36^B^	0.43^A^
	Montana	351^C^	362^B^	269^C^	0.56^C^	3.7^E^	266^C^	27.3^C^	0.18^E^	0.46^A^
	Illinois	370^B^	386^A^	231^D^	0.60^B^	3.9^DE^	228^D^	23.5^C^	0.25^D^	0.48^A^
*Amaranthus hypochondriacus*	India	354^C^	281^E^	346^A^	0.57^C^	6.3^A^	340^A^	35.2^A^	0.31^C^	0.42^A^
	Nebraska	325^D^	309^D^	348^A^	0.50^D^	5.0^B^	343^A^	35.3^A^	0.40^A^	0.45^A^
	Pennsylvania	382^A^	285^E^	311^B^	0.64^A^	4.4^CD^	307^B^	31.6^B^	0.30^C^	0.34^B^
RMSE		0.78	2.42	0.71	0.00006	0.002	0.69	0.075	0.0007	0.003

The concentration of fatty acids was expressed as g kg^−1^, considering 1 kg the sum of the areas of all FAME identified. SFA = saturated fatty acids; MUFA = monounsaturated fatty acids; PUFA = polyunsaturated fatty acids; n3 = n3‐polyunsaturated fatty acids; n6 = n6‐polyunsaturated fatty acids; SFA/UFA = saturated/unsaturated fatty acid ratio; PI = peroxidation index; TPC = total phenolic content expressed as gallic acid equivalents (GAE mg g^−1^ seeds); DPPH = scavenging activity expressed as EC_50_ (concentration of dried extract mg mL^−1^ solution). RMSE: root mean square error; down the column, lower case and upper case letters indicate *P* < 0.01 and *P* < 0.05, respectively.

### *In vitro* fermentation characteristics, kinetics and end‐products

**Figure 4:**
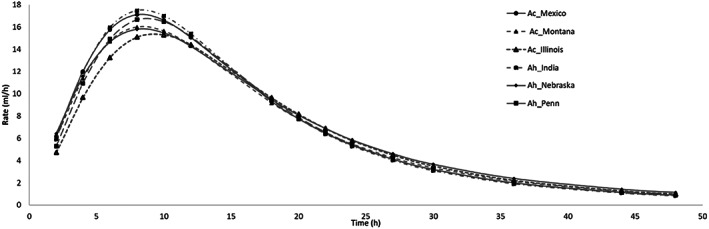
*In vitro* fermentation rate over time in the studied accessions of the two amaranth species. Ac: *A. cruentus*, Ah: *A. hypochondriacus*.

Table 4 shows the characteristics and kinetics of *in vitro* fermentation for the six accessions of amaranth seed. Only a few differences appeared among all the accessions, only Illinois showed the significantly highest (*P* < 0.01) OMD and OMCV values. For kinetics parameters, the Illinois and Nebraska accessions showed higher values of *T*/2 (*P* < 0.01) than the other accessions; Nebraska also showed the lowest C value (*P* < 0.01). The rate of fermentation is depicted in Fig. 4 where it is more evident that all samples showed a similar curve profile: a rapid increase, maximum rate after 8 h, a slow decrease with a stop around 24 h. However, Illinois showed a fermentation process slower and Mexico faster compared to the other ones.

**Table 4:** *In vitro* fermentation characteristics in the six accessions of amaranth seeds (*n* = 18)

Species	Accessions	OMD (%)	OMCV (mL g^−1^)	*B* (h)	*C*
*Amaranthus cruentus*	Mexico	91.7^B^	318^B^	13.5^B^	2.12^A^
	Montana	92.8^B^	315^B^	13.1^B^	2.19^A^
	Illinois	99.5^A^	350^A^	14.7^A^	2.16^A^
*Amaranthus hypochondriacus*	India	93.1^B^	322^B^	13.3^B^	2.17^A^
	Nebraska	91.0^B^	321^B^	14.6^A^	1.99^B^
	Pennsylvania	91.2^B^	313^B^	13.7^B^	2.19^A^
RMSE		1.83	45.6	0.46	0.05

OMD: organic matter disappearance; OMCV: cumulative volume of gas related to incubated organic matter; *B*: the time at which half of the asymptote is reached; *C*: the switching characteristic of the curve.

RMSE: root mean square error. Down the column upper case letters indicate *P* < 0.01.

Table 5 shows the *in vitro* fermentation end products for the six accessions of amaranth seed. The pH values had a limited variation and the Illinois and India accessions showed the highest values, whereas the Montana accession had the lowest. The Nebraska accession presented the highest amount of total volatile fatty acids while the Illinois accession had the lowest one (*P* < 0.05). No significant differences regarding the proportion of the single volatile fatty acids were observed between accessions. Mexico showed the highest proportion of BCFA in comparison with Montana and Pennsylvania (*P* < 0.05).

**Table 5:** Fermentation end‐products in the six accessions of amaranth seeds (*n* = 18)

Species	Accession	pH	Ace	Prop	Iso‐But	But	Iso‐Val	Val	tVFA	BCFA	Ace/Prop
			g 100 g^−1^ tVFA						mmol g^−1^ OM	g 100 g^−1^ tVFA	
*Amaranthus cruentus*	Mexico	6.54^ab^	57.1	22.3	0.96	16.1	2.54	1.53	92.3^ab^	3.82^a^	2.57
	Montana	6.46^b^	58.1	20.8	0.83	18.0	1.79	1.27	97.2^ab^	2.62^b^	2.72
	Illinois	6.59^a^	58.0	21.6	0.91	16.0	2.10	1.30	89.1^b^	2.93^ab^	2.73
*Amaranthus hypochondriacus*	India	6.55^a^	56.9	23.6	0.96	15.1	2.16	1.45	92.5^ab^	3.12^ab^	2.52
	Nebraska	6.52^ab^	55.8	22.3	0.90	17.7	2.10	1.56	99.9^a^	3.37^ab^	2.42
	Pennsylvania	6.54^ab^	54.3	23.3	0.92	17.3	1.87	1.38	96.2^ab^	2.68^b^	2.36
RMSE		0.001	1.32	1.05	0.15	0.88	0.18	0.11	5.52	0.07	0.02

Ace: acetate; Prop: propionate; Iso‐But: iso‐butyrate; But: butyrate; Iso‐Val: iso‐valerate; Val: valerate; tVFA: total volatile fatty acids;

BCFA: Branched chain fatty acids (Iso‐But + Iso‐Val/tVFA × 100); Ace/Prop: acetate/propionate;

RMSE: root mean square error. Down the column uppercase letters indicate *P* < 0.05.

### Correlation

Significant correlations were observed when comparing *in vitro* parameters, fatty acid classes, the quality index, and antioxidant properties (Table 6). In particular, MUFAs, PUFAs and n‐6 were significantly correlated with OMCV and rate of fermentation, whilst SFAs were negatively related (*P* < 0.05) with both branched fatty acids production.

**Table 6:** Correlation between *in vitro* parameters, fatty acid classes, quality index, and antioxidant properties

	OMCV	*R*_max_	Ace	Prop	Iso‐but	Iso‐Val
SFA	0.253	−0.376	−0.543	−0.515	−0.653	−0.589
	NS	NS	NS	NS	*	*
MUFA	0.575	−0.643	−0.041	−0.334	−0.187	−0.210
	*	*	NS	NS	NS	NS
PUFA	−0.617	0.736	0.333	0.528	0.485	0.463
	*	**	NS	NS	NS	NS
n‐3	−0.182	0.389	−0.048	0.228	0.254	0.237
	NS	NS	NS	NS	NS	NS
n‐6	−0.623	0.740	0.339	0.531	0.488	0.466
	*	**	NS	NS	NS	NS
PI	−0.610	0.732	0.327	0.524	0.483	0.461
	*	**	NS	NS	NS	NS
TPC	−0.183	0.262	0.203	0.582	0.640	0.701
	NS	NS	NS	*	*	**
DPPH^•^	0.587	−0.475	0.296	−0.258	−0.005	−0.147
	*	NS	NS	NS	NS	NS

OMCV: cumulative volume of gas related to incubated organic matter; R_max_: maximum fermentation rate; Ace: acetate; Prop: propionate; Iso‐But: iso‐butyrate; Iso‐Val: iso‐valerate; SFA: saturated fatty acids; MUFA: monounsaturated fatty acids; PUFA: polyunsaturated fatty acids; n3: n‐3 polyunsaturated fatty acids; n6: n‐6 polyunsaturated fatty acids; PI: peroxidation index; TPC: total phenolic content; DPPH: radical scavenging activity. *, **, and NS indicate *P* < 0.05, *P* < 0.01, and not significant, respectively.

For SFA, a significant (*P* < 0.05) negative correlation was found with myristic acid (C14:0), propionate (*r =* −0.6449), iso‐butyrate (*r =* −0.5832), and iso‐valerate (*r =* −0.5839), and between palmitic acid (C16:0) and iso‐butyrate (*r =* −0.5863); among the long‐chain fatty acids, the stearic acid (C18:0) showed a significant negative (*P* < 0.05) correlation (*r =* −0.6231) with propionic acid. Among the unsaturated fatty acids (MUFAs and PUFAs) in amaranth seeds, oleic (C18:1n9), linoleic (C18:2n6), and linolenic (C18:3n3) acids did not affect the amount of acetate, propionate, iso‐butyrate, and iso‐valerate (data not reported).

Regarding antioxidant properties, the peroxidation index showed a significant (*P* < 0.05) negative correlation with gas production (*r =* −0.6103; *P* < 0.05) and positive with *R*_max_ (*r =* 0.7323; *P* < 0.01); the DPPH^•^ scavenging activity showed a significant (*P* < 0.05) positive correlation with gas production (*R =* 0.5867). Total phenolic content was correlated (*P* < 0.05) with propionate, iso‐butyrate, and (*P* < 0.01) iso‐valerate.

## DISCUSSION

### Agronomic aspects

Plant height is an important parameter in the combine harvesting efficiency of grain crops. Short plants will generate grain loss, while plants that are too high will produce great biomass at the expense of grain production. In our trial, all amaranth accessions of both species at harvest were within the optimal height range for mechanical harvest (from 117 to 140 cm): slightly higher than traditional cereals but shorter than the ranges reported by Casini and La Rocca^30^ and El Gendy *et al*.^31^ for the same species. The seed yields of *A. hypochondriacus* and, above all, of *A. cruentus*, were very interesting and generally higher than those reported in other research in central Italy.^30^, ^31^ Seed production was also higher than that obtained by Gresta *et al*.^32^ in the same environment, for the same species, but with different accessions, the recorded yields of which ranged from 2.2 to 2.8 t ha^−1^. As a whole, our yield was comparable with those of the main traditional cereal species grown in the Mediterranean environment. The thousand seed weight showed a limited variability, in agreement with that reported by Gimplinger *et al*.,^33^ confirming that seed yield does not depend on the unitary seed weight but it mainly depends on the number of seeds produced.

### *In vitro* fermentation pattern

According to other *in vitro* studies, carried out on more conventional feedstuffs,^34^, ^35^ the fermentation characteristics of amaranth grains were affected by their chemical composition. However, considering the relatively high lignin content recorded for all amaranth seeds, the high OMD values could be ascribed to filtration problems, as previously observed when substrates rich in high soluble carbohydrates have been analyzed.^36^

In particular, ether extract was inversely related to OMCV (*r =* −0.8452, *P* < 0.001) and NDF was directly correlated with total VFA (*r =* 0.7123; *P* < 0.01) (data not shown). Adequate fermentation is also shown by the pH values recorded at the end of the incubation period (from 6.46 to 6.59), which ensured a favorable environment for the activity of cellulolytic bacteria in the rumen. In general, few differences emerged between species and accessions. Only Illinois differs from the others, showing nonlinear results: it had the best performance in terms of gas volume but the lowest VFA production and the slowest fermentation kinetics. Some studies reported the possible utilization of amaranth grain in ruminant diets in substitution of traditional cereal.^13^ Jalč *et al*.,^11^ using an artificial rumen, evaluated the effect of *A. hypochondriacus* as a partial substitution (5, 10, and 20%) for barley. The VFAs, methane, ammonia and total gas production, organic matter, and fiber digestibility were similar at all the dosages considered. Kubelková *et al*.,^12^ using rumen simulation technique (RUSITEC), observed no effect on DM and acid detergent fibre (ADF) degradability, total gas and VFA in ruminant diet replacing (10%) barley with amaranth seeds. The authors only found a reduction in degradability for NDF. There has been little research on the incubation of seeds alone in the literature.

On average, the *in vitro* fermentation parameters of the studied *Amaranthus* accessions were comparable with other concentrates traditionally used in ruminant diets; they showed high degradability and fermentability and fast kinetics: OMD 93.21%, OMCV 323 mL g^−1^, *R*_max_ 16.7 mL h^−1^, *T*_max_ 8.60 h. These data are close to those reported in a previous *in vitro* study^37^ incubating cereal with dairy cow inoculum: barley meal (OMD 95.44%; OMCV 365 mL g^−1^; *R*_max_ 13.70 mL h^−1^, *T*_max_ 18.0 h) and corn meal (OMD 88.91%; OMCV 387 mL g^−1^; *R*_max_ 19.6 mL h^−1^, *T*_max_ 6.78 h). The production of the main VFA was quite similar to that observed by incubating barley and corn meal, demonstrating that, for ruminant nutrition, *Amaranthus* could be considered a valid alternative to cereal grains. Considering other nutrients of *Amaranthus* seeds (i.e., protein, lipids) we compared the results of this study with the *in vitro* fermentation characteristics of some legume grain previously studied^38^, ^39^ using bovine inoculum. Similar results emerged for cumulative gas production with faba beans (320 mL g^−1^) and lupine (317 mL g^−1^), while amaranth OM disappearance was higher than that of both legume grains (85% and 89%, respectively).

### *In vitro* fermentation versus fatty acid profiles

The PUFAs, specifically those of the n6 series represented by the linoleic acid (C18:2n6), reduced the production of gas in the rumen, whereas the MUFAs, specifically oleic acid (C18:1n9), positively affected OMCV. The maximum fermentation rate was positively affected by PUFA and negatively by MUFA. A possible explanation for the effects of unsaturated fatty acids on *in vitro* fermentation characteristics and end products could be that the extent of protection of the fatty acids in amaranth seed oil resulted in a release of fatty acids into the rumen fluid at a high rate, which inhibited the hydrogenation of the PUFAs but, at the same time, allowed a high concentration of these fatty acids in the rumen, increasing the effects on the digestion of organic matter.^40^ These observations are also in agreement with those of Ikwuegbu and Sutton,^41^ who explained that the intensity of fermentation in the rumen depends on the quantity and quality of fatty acids.^40^, ^42^

Medium‐ and long‐chain free fatty acids, released by hydrolysis in the rumen, may alter the synthesis of lipids by rumen microbes.^43^, ^44^ In our study, the fatty acid profile of amaranth seeds had no effect on the concentration of total VFA (data not shown), as observed by Kubelková *et al*.^12^ studying the effect of dietary substitution of 10% of barley meal with *A. hypochondriacus* seeds on fermentation parameters in an artificial rumen.

However, changes in the proportions of individual VFA (acetic, propionic, iso‐butyric and iso‐valeric acids) were found in some saturated fatty acids (C14:0, C16:0, C18:0), in agreement with the observations of Sutton *et al*.^40^ regarding the rumen of sheep fed free lipid supplement. However, the high proportion of unsaturated fatty acids (MUFAs and PUFAs) in amaranth seeds did not affect the amount of acetic, propionic, iso‐butyric, and iso‐valeric acids. This is in contrast with the McAllister *et al*.^45^ observations on the toxic effect of long‐chain unsaturated fatty acids on protozoa, methanogens, and cellulolytic bacteria in the rumen. Nevertheless, the same authors found that propionate‐producing bacteria were insignificantly inhibited by long‐chain fatty acids in agreement with our results. It is possible to hypothesize a sort of protection of the fatty acids in amaranth seed oil, which reduces the effects of the oil on rumen volatile fatty acids and microbial synthesis, as observed by Sutton *et al*.^40^ Furthermore, it is also possible that the phenolic compounds of amaranth seeds complexed with the oils inhibited the effect of long‐chain unsaturated fatty acids on VFA.^46^

### *In vitro* fermentation versus phenolic compounds and DPPH

According to Vasta *et al*.,^47^ the phenolic compounds influence the ruminal microbiota, modulating the environment and fermentations. Polyphenols interfere with the ruminal biohydrogenations causing a positive effect on long‐chain fatty acid production,^48^, ^49^ even though analysis of microbiota composition did not provide clear indications about which changes in bacteria population are responsible for the effects on rumen biohydrogenation.^47^ Early studies on the effect of dietary polyphenols showed a general depressive effect of these compounds on rumen microbiota, resulting in a reduction in VFA production. Recent *in vitro* ^50^, ^51^ and *in vivo* ^52^, ^53^, ^54^, ^55^ studies on VFA production, using new‐generation molecular techniques, showed a positive effect of polyphenols on propionate and, in some cases, on total VFA production. Our results agree with these observations, showing a significant positive correlation of total phenolic content with individual VFAs (acetic, propionic, iso‐butyric and iso‐valeric acids). Probably this effect could be related to the nature of polyphenols,^47^ which in *A. cruentus* seeds are represented by caffeic, ferulic, sinapic, *p*‐coumaric, cinnamic, *p*‐hydroxybenzoic, and vanillic acids,^56^ whereas, as far as we are aware, no data are reported on the phenolic profile of *A. hypochondriacus* seed.

A high peroxidation index, related to the degree of fat saturation, increases the rate of rumen fermentation but reduces the fermentation efficiency, even if, to date, the optimal fat saturation rate that reduces the inhibitory effect on ruminal function has not been established.^57^ Data obtained on the peroxidation index showed that the *in vitro* dietary inclusion of amaranth seeds in dairy cows seemed to interfere with microbial populations and ruminal processes without modifying the fermentation end‐product profile. As Palmquist and Jenkins^57^ speculated, an increase in forage in the diet could dilute the negative effects of unsaturated fatty acids of amaranth seed oil.

As regards the DPPH^•^ scavenging activity, our results showed that a decrease in antioxidant activity caused an increase in the disappearance of organic matter and in the cumulative volume of gas related to incubated organic matter. From a nutritional point of view, the decrease in the antioxidant level may impair the health and performance of ruminants through an oxidant‐antioxidant imbalance^58^ but, at the same time, an increase in the OMD could facilitate the achievement of optimal ruminal availability of energy and thus a more efficient use of energy in ruminant diets.^59^

## CONCLUSIONS

The present research proved that *A. cruentus* and *A. hypochondriacus* can be grown in the Mediterranean environment with excellent results comparable with, or higher than, those of the traditional cereal crops in this environment. From a health point of view, the Nebraska accession of *A. hypochondriacus* represented the best accession, showing the lowest saturated fatty acid level and the highest polyunsaturated fatty acid content. Considering the nutritive value, few differences appear between species and accessions; nevertheless, the Illinois accession showed the best performance in terms of gas volume but not for the VFA production for which it showed the lowest value. Regarding kinetics, the Illinois accession showed the slowest fermentation, whereas the Montana and India accessions had the fastest ones.

Further studies are required to explain better the influence of fatty acids on specific mechanisms involved in ruminal microbiota turnover in the digestive process in the rumen and to explain the exact effects of the antioxidant activity on the rumen's metabolism.

## ETHICAL APPROVAL

This article does not contain any studies with human or animal subjects.
